# The prevalence and intensity of external and internal parasites in working donkeys (*Equus asinus*) in Egypt

**DOI:** 10.14202/vetworld.2018.1298-1306

**Published:** 2018-09-19

**Authors:** Marwa M. Attia, Marwa M. Khalifa, Marwa Th. Atwa

**Affiliations:** 1 Department of Parasitology, Faculty of Veterinary Medicine, Cairo University, Giza, Egypt; 2 Department of Zoology, Faculty of Science, Al-Fayoum University, Egypt

**Keywords:** arthropods, donkeys, Egypt, external parasites, helminths, internal parasites, protozoa

## Abstract

**Aim:**

This study aims to record and update the prevalence and intensity of external and internal parasites in working donkeys (*Equus asinus*) in Egypt during the period from January to December 2017.

**Materials and Methods:**

A total of 120 donkeys (10 donkeys each month) were examined at Giza zoo abattoir through bimonthly visits. The examined donkeys were obtained from five governorates (Giza [20], Fayoum [40], Beni Suef [30], Monofia [20], and Assiut [10]). The animals were grouped according to age and sex.

**Results:**

All examined donkeys were positive with at least one internal or even external parasitic species. The overall prevalence rate was 100%. A total of 11 helminths species (10 nematodes and 1 metacestode); 7 protozoal and 7 arthropod species were collected. The number of each parasite and intensity of infection with regard to age and sex was recorded.

**Conclusion:**

All examined donkeys were infected with parasites with an overall prevalence of 100%. So, we recommended following up and continuous treatment of such diseased animal.

## Introduction

More than 40 million donkeys are distributed throughout the world [1]. The donkey population in Africa is estimated to be 13 million [2]. According to the latest Food and Agriculture Organization statistics, there are approximately 3 million working donkeys in Egypt. The working donkeys, horses, and mules carry out a wide range of work types. These animals are used for transportation of passengers and goods by carts in urban areas in the busy cities and towns.

The most important problems for equines and donkeys in developing countries are gastrointestinal parasitism [3]. Donkeys harbor a large number of parasites including roundworms (families: *Stronglidae, Oxyuridae, Trichostronglidae*, and *Ascaridae*), flatworms (*Fasciolidae*), and tapeworm (family: *Anoplocephalidae*) which damage the intestine depending on the species and number of parasites [3]. Infections with endoparasites cause loss of condition, poor reproduction of animals, colic, and diarrhea [4].

Furthermore, blood protozoal diseases are one of the important parasitic infections which affect family Equidae in Egypt. Equine piroplasmosis is the tick-borne disease caused by *Theileria equi* (*Babesia equi*) which causes abortions, loss of performance, and death [5]. Trypanosomes are blood parasites found in mammals including donkeys; *Trypanosoma evansi* which is one of the trypanosomes infecting donkeys [6].

This study aims to record and update the prevalence and intensity of external and internal parasites in working donkeys (*Equus asinus*) in Egypt during the period from January to December 2017.

## Materials and Methods

### Ethical approval

This study was approved by the Ethical Committee, Faculty of Veterinary Medicine, Cairo University with number CU/II/F/18/103.

### Animals

During the period from January to December 2017, 120 donkeys (10 donkeys each month) were examined at postmortem in Giza Zoo abattoir (Giza, Egypt) through bimonthly visits, for the detection of internal and external parasitic infection. The donkeys were obtained from five governorates (Giza [20], Fayoum [40], Beni Suef [30], Monofia [20], and Assiut [10]). The animals were grouped according to age as from 1 to 2 years (25), 3-5 years (35), and 6-8 years (60), of which 90 donkeys were male and 30 were female. The animals were field working donkeys, fed on green ration, and never received any antiparasitic medications. These animals send for slaughtering in this abattoir are usually emaciated and unsuitable for working. Each donkey was physically examined before slaughtering, for determination of the age and sex as well as examination of external parasites on skin.

### Fecal sample collection and examination

Fecal samples were collected directly from the rectum of donkeys before slaughtering. The feces were collected in separate polyethylene bags and labeled for identification. Microscopic examination of the samples was performed in the Laboratory of the Parasitology Department in the Faculty of Veterinary Medicine, Cairo University, Giza, Egypt. The gross fecal examination was done for the collection of adult nematodes and/or the gravid segment of cestodes.

### Microscopic fecal examination

#### Direct smear method

A small amount of feces was placed on the clean glass slide and mixed with a drop of water; a coverslip was applied on the fecal smear and examined under the microscope to detect and identify the parasitic ova [7].

### Floatation and sedimentation technique

#### Floatation technique

One g of feces was diluted with 10 ml of saturated salt solution in the test tube which was filled to the top with the salt. A clean cover glass slip was sideways over the top of the tube. After 10 min, the cover was taken onto the slide and examined under the light microscope using the magnification power 40 and 100×.

#### Sedimentation method

Two g of feces was dissolved in tap water in a beaker and allowed the mixture to sediment without disturbing for 20-30 min. The supernatant was poured off to collect the sediment for examination [7]. A small amount of the sediment was transferred to a small Petri dish and examined under the light microscope using the magnification power 40 and 100×.

### Examination of gastrointestinal samples

The samples were collected from stomach and small and large intestine after slaughtering the donkeys for detection of parasites as following:

Stomach and intestinal contents from every donkey were examined separately by naked eyes, the larvae and adult worms were collected. The collected samples were placed in a separate vial containing a saline solution (0.9% NaCl). The wall of the stomach and intestine were washed separately, and these washings were collected for subsequent examination. All of the collected helminths were preserved in 70% glycerol alcohol for subsequent identification. The stomach larvae (*Gasterophilus* spp.) were collected in 70% ethanol until identification study.

Smears were made from each intestinal sample of different parts of the intestine for examination of *Cryptosporidium* species. Each sample was mixed thoroughly with the drop of saline and spread on glass slides which left to air dry at the room temperature, fixed by absolute methanol for 10 min and stained with modified Ziehl–Neelsen stain technique [8]. Other smears were made and also fixed in absolute methanol which stained with Giemsa stain for examination of other enteric protozoa.

### Examination of tissue for detection of *Sarcocystis* spp.

Samples from esophagus, heart, tongue, and diaphragm were fixed in 10% formalin and processed as recorded by Bancroft and Stevens [9]. Sections were deparaffinized and stained with hematoxylin and eosin stain for histological examination by light microscopy.

### Blood samples

Blood samples were collected directly from the jugular vein into heparinized test tubes at the time of slaughtering. Thin blood smears were made and left to air dry. The smears were fixed with absolute methanol and stained with Giemsa stain. Slides were examined under a microscope using the oil immersion lens for the identification of blood parasites [7].

### External parasites

The skin was carefully examined for the presence of any external parasites such as ticks and any insect’s flies which were identified using a stereoscopic microscope. Skin scraping was done if keratinization was present in the skin according to Soulsby [7].

### Cellophane tape technique

To detect the eggs of pinworms (*Oxyuris equi*), female nematodes were protruded from the anus and deposited their eggs on the skin around the anus. The cellophane tape was used around the anus and then placed it on the slide with the small drop of water and examined under a light microscope with magnification X40, X100, and X400.

### Identification of the parasites

The nematodes were washed several times with phosphate buffer saline (pH 7.2), then preserved in 70% glycerin alcohol. The nematodes were cleared using lactophenol, then mounting by gelatin. All identifications of the helminths and their eggs were carried out following the morphological description [7,10-12]. All arthropod larvae, fleas, ticks, and mites, as well as all protozoan parasites, were identified according to Soulsby [7].

### Statistical analysis

The prevalence of infection and intensity was calculated using Chi-square test, with determination of mean intensity related to governorates [13]. Significance was analyzed using the SPSS v.11.0. In all cases, p<0.05 were considered for the statistically significant difference.

## Results

**Figure-1:**
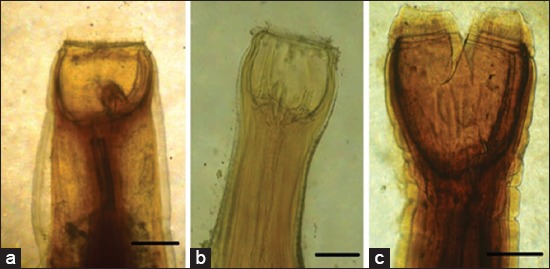
Strongylus spp. infecting large intestine of donkeys (notes its buccal capsules). (a) *Strongylus vulgaris* (two ear-shaped subdorsal teeth), (b) *Strongylus equinus* (three teeth; one large bifid teeth and two smaller one), (c) *Strongylus edentatus* (buccal capsules with no teeth), Scale bar 100 μm.

**Figure-2:**
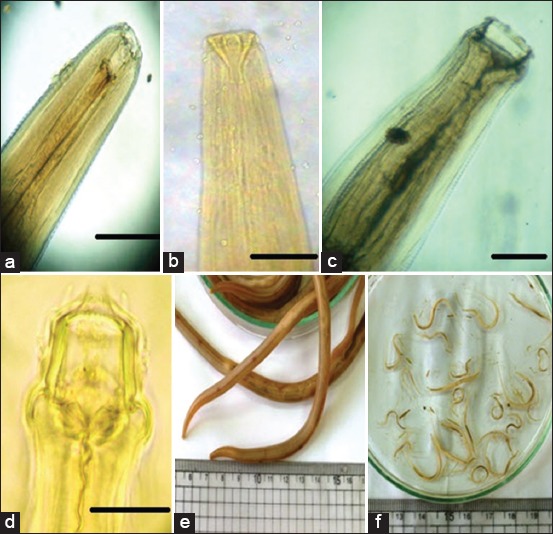
(a) *Habronema muscae* (cylindrical pharynx), (b) *Habronema megastoma* (funnel-shaped pharynx), (c) *Cylicocyclus asini*, (d) *Cyathostomum* spp., (e) *Parascaris equorum* (large lips), (f) *Oxyuris equi* (pinworm).

**Figure-3:**
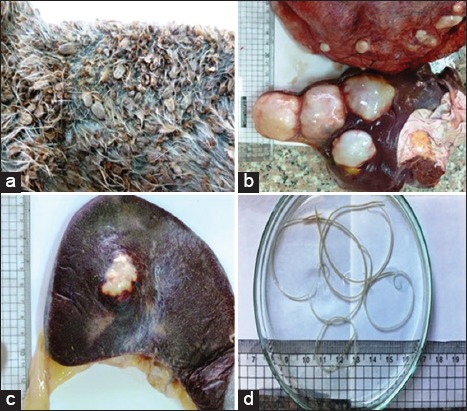
(a) Skin heavily infested with ticks, (b and c) hydatid cyst in liver, lung, and spleen, (d) *Setaria equina* (filarial nematodes of equines from the peritoneal cavity).

**Figure-4:**
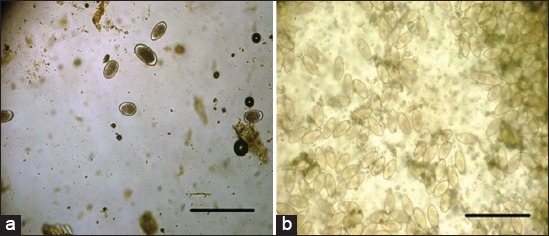
(a) Strongylus eggs, (b) *Oxyuris equi* eggs with cellophane tape techniques, Scale bar 100 μm.

All the donkeys examined were positive with at least with one internal or external parasitic species. The overall prevalence rate was 100%. A total of 11 helminths species (10 nematodes and 1 metacestode) were recorded. Higher prevalence was estimated in *Cylicocyclus asini* (91.66%) followed by *Cyathostomum* spp. (83.33%) while lower prevalence recorded in *Draschia megastoma* and hydatid cyst (8.33%). The helminths species and their prevalence are given in Table-1 and Figures-1, 2, 3b-d, and 4.

**Table-1:** Prevalence and mean intensity of helminth parasites infecting donkeys.

Parasites	No. infected (%)	Range (intensity)	Sex		Age		
	
			M (%)	F (%)	1-2	3-5	6-8
*Habronema muscae*	90 (75)	50-300 (80)	65 (72.22)	25* (83.33)	3 (12)	32** (91.42)	55 (91.66)
*Draschia megastoma*	10 (8.33)	2-10 (7)	7 (7.77)	3 (10)	-	4 (11.42)	6* (10)
*Parascaris equorum*	30 (25)	15-40 (25)	24 (26.66)	6** (20)	25 (100)	5** (14.28)	-
*Strongylus vulgaris*	20 (16.66)	1-110 (30)	9 (10)	11 (36.66)	-	8 (22.85)	12 (20)
*Strongylus equinus*	30 (25)	2-45 (15)	13 (14.44)	17 (56.66)	-	12 (34.28)	18 (30)
*Strongylus edentatus*	30 (25)	2-55 (20)	12 (13.33)	18 (60)	-	10 (28.57)	20* (33.33)
*Cyathostomum* spp.	100 (83.33)	20-100 (50)	85 (94.44)	15** (50)	12 (48)	28* (80)	60 (100)
*Cylicocyclus asini*	110 (91.66)	5-26 (15)	80 (88.88)	30* (100)	20 (80)	30 (85.71)	60 (100)
*Oxyuris equi*	60 (50)	7-30 (10)	34 (37.77)	26 (86.66)	18 (72)	30* (85.71)	12 (20)
*Setaria equina*	30 (25)	1-15 (5)	12 (13.33)	18 (60)	-	12 (34.28)	18 (30)
Hydatid cyst	10 (8.33)	1-3 (1)	4 (4.44)	6 (20)	-	3 (8.57)	7 (11.66)

* p≤0.05;

** p≤0.01, M: Male, F: Female

**Figure-5:**
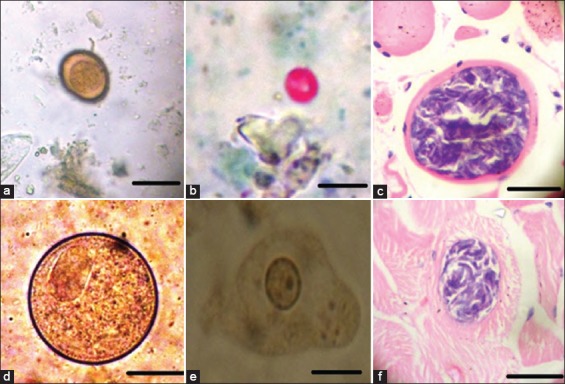
Protozoan parasites infection donkeys. (a) *Eimeria leuckarti*, (b) *Cryptosporidium* spp., (c and f) *Sarcocystis* spp. in muscles and heart, (d) *Balantidium coli*, (e) *Entamoeba coli* vegetative form. Scale bar 50 μm.

**Figure-6:**
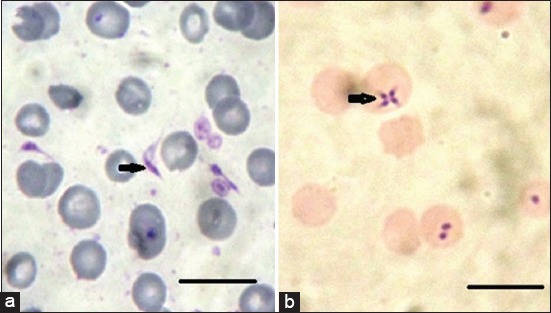
(a) *Trypanosoma evansi*, (b) *Theileria equi*; blood smears stained with Giemsa stain. Scale bar 10 μm.

A total of seven protozoal species were recorded with the most prevalent one being *Balantidium coli* with 91.6% and the lowest prevalent one being *Cryptosporidium* spp. 6.66% which was present mainly in young donkeys aged between 1 and 2 years and become lower at the older age. The rates of *Sarcocystis* infection in the esophagus, tongue, diaphragm, and heart were 80%, 97%, 40.0%, and 14%, respectively. The rates of detection by age were as follows: 1-2 years old 17%, 3-5 years old 45%, and 6-8 years old 50% (Table-2 and Figures-5 and 6).

**Table-2:** Prevalence of protozoal infection in donkeys (n=120) with reference to sex and age.

Protozoa	No. infected	Sex		Age		
	
		M (%)	F (%)	1-2 (%)	3-5 (%)	6-8 (%)
*Eimeria leuckarti*	10 (8.33)	8 (8.88)	2 (6.66)	-	6* (17.14)	4 (6.66)
*Cryptosporidium* spp.	8 (6.66)	6 (6.66)	2 (6.66)	6 (24)	2* (5.71)	-
*Sarcocystis* spp.	80 (66.66)	60 (66.66)	20** (66.66)	-	30 (85.71)	50 (83.33)
*Balantidium coli*	110 (91.66)	90 (100)	20** (66.66)	20 (80)	30 (85.71)	60* (100)
*Entamoeba coli*	30 (25)	26 (28.88)	4* (13.33)	3 (12)	20 (57.14)	7** (11.66)
*Theileria equi*	20 (16.66)	16 (17.77)	4 (13.33)	-	8 (22.85)	12 (20)
*Trypanosoma evansi*	16 (13.33)	12 (13.33)	4 (13.33)	-	6 (17.14)	10 (16.66)

* p≤0.05;

** p≤0.01, M: Male, F: Female

**Figure-7:**
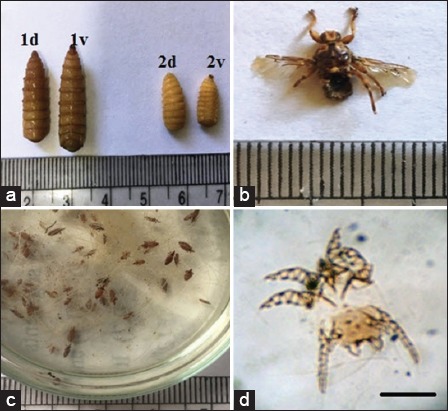
Ectoparasites infesting donkeys. (a) *Gasterophilus intestinalis* (1) 1d on dorsal view, 1v on ventral view, 2: *Gasterophilus nasalis*, 2d on dorsal surface; 2v on ventral surface. (b) *Hippobosca equina*, (c) *Haematopinus asini*, (d) *Psoroptes equi*, scale bar100 μm.

The findings on the arthropods in this study include 7 species were recorded with highest infestation rate in *Gasterophilus intestinalis* (97.5%) and lower infestation rate recorded in *Haematopinus asini* and *P. equi* (8.33%) (Table-3 and Figures-3a and 7).

**Table-3:** Prevalence and mean intensity of arthropod parasites infected donkeys.

Arthropods	No. infected (%)	Sex		Age		
	
		M (%)	F (%)	1-2	3-5	6-8
*Gasterophilus intestinalis*	117 (97.5)	87 (96.66)	30** (100)	22 (88)	35 (100)	60* (100)
*Gasterophilus nasalis*	80 (66.66)	68 (75.55)	12** (40)	-	20 (57.14)	608 (100)
*Boophilus* spp.	12 (10)	10 (11.11)	2* (6.66)	-	2 (5.7)	10 (16.66)
*Hippobosca equina*	15 (12.5)	7 (7.77)	8 (26.66)	-	6 (17.14)	9 (15)
*Haematopinus asini*	10 (8.33)	4 (4.44)	6 (20)	1 (4)	4 (11.42)	5 (8.33)
*Ctenocephalides felis*	70 (58.33)	47 (52.22)	23* (76.66)	15 (60)	25 (71.42)	30 (50)
*Psoroptes equi*	10 (8.33)	7 (7.77)	3 (10)	-	4 (11.42)	6 (10)

* p≤0.05;

** p≤0.01, M: male, F: female

As for the sex in our study, males were high in *B. coli* (100%) followed by *G. intestinalis* (96.66%) and *C. asini* (88.88%). The prevalence in males was lower in *Cryptosporidium* spp. (6.66%), *H. asini* (4.44%), and hydatid cyst (4.44%).

The prevalence in females was higher in *G. intestinalis*, *C. asini* (100%), *Sarcocystis* spp., and *B. coli* (66.66%), while lower in *Rhipicephalus* spp., *Eimeria leuckarti, Cryptosporidium* spp. (6.66%), and *D. megastoma* (10%).

With regard to age in this study, the age from 1 to 2 years was higher in *Parascaris equorum* (100%), *G. intestinalis* (88%), and *B. coli* (80%) and lower in *Entamoeba coli*, *Habronema muscae* (12%), and *H. asini* (4%). In age ranged from 3 to 5 years, *G. intestinalis* (100%), *H. muscae* (90%), *Sarcocystis* spp., *B. coli*, and *C. asini* (85.71%) and lower in *Cryptosporidium* spp., *Boophilus* spp. (5.71%), and hydatid cyst (8.57%). In age ranged from 6 to 8 years, *G. intestinalis, Gasterophilus nasalis*, *B. coli*, *Cyathostomum* spp., and *C. asini* were 100%, while in *E. leuckarti* (6.66%), *H. asini* (8.33%), and *D. megastoma* (10%).

Regarding geographical distribution of parasitic infection in Egyptian donkeys, the four governorates were positive for single or mixed infection. The Giza governorate was higher in *C. asini* (75%), *B. coli* (80%), and *G. intestinalis* (90%) and lower in *S. equina*, *T. equi*, and *P. equi* (5%).

The prevalence rate in Fayoum governorate was higher in *H. muscae*, *Cyathostomum* spp., *C. asini*, and *G. intestinalis* (100%) and lower in *Cryptosporidium* spp. and *P. equi* (12.5%).

The prevalence rate in Beni Suef governorate was higher in *G. intestinalis* (100%) and lower in hydatid cyst and *Cryptosporidium* spp. (3.33%). While in Monofia the highest prevalence rate of infestation was recorded in *G. intestinali*s (95%) and *C. asin*i (90%) and lower in *D. megastom*a, *S. equin*a, and *P. equ*i (5%). The prevalence studies in Assiut governorate were higher in *Cyathostomum* spp., *B. coli, T. evansi*, and *G. intestinalis* (100%), and lower prevalence was recorded in *D. megastoma* (10%). The geographical distribution of each parasite in examined governorates is recorded in Table-4.

**Table-4:** Prevalence of infection by different parasites in examined governorates.

Parasites	Number of positive donkeys (%)					

	Governorates	Giza n=20	Fayoum n=40	Beni Suef n=30	Monofia n=20	Assiut n=10
Helminths	*Habronema muscae*	10 (50)	40 (100)	23 (76.66)	9 (45)	8 (80)
	*Draschia megastoma*	-	6 (15)	2 (6.66)	1 (5)	1 (10)
	*Parascaris equorum*	-	20 (50)	2 (6.66)	3 (15)	5 (50)
	*Strongylus vulgaris*	-	15 (37.5)	2 (6.66)	-	3 (30)
	*Strongylus equinus*	-	20 (50)	4 (13.33)	1 (5)	5 (50)
	*Strongylus edentatus*	-	21 (52.5)	5 (16.66)	2 (10)	2 (20)
	*Cyathostomum* spp.	10 (50)	40 (100)	27 (90)	13 (65)	10 (100)
	*Cylicocyclus asini*	15 (75)	40 (100)	28 (93.33)	18 (90)	9 (90)
	*Oxyuris equi*	2 (10)	25 (62.5)	20 (66.66)	8 (40)	5 (50)
	*Setaria equina*	1 (5)	20 (50)	2 (6.66)	-	7 (70)
	Hydatid cyst	-	7 (17.5)	1 (3.33)	-	2 (20)
Protozoa	*Eimeria leuckarti*	-	7 (17.5)	3 (10)	-	-
	*Cryptosporidium* spp.	-	5 (12.5)	1 (3.33)	-	2 (20)
	*Sarcocystis* spp.	3 (15)	37 (92.5)	26 (86.66)	5 (25)	9 (90)
	*Balantidium coli*	16 (80)	39 (97.5)	28 (93.33)	17 (85)	10 (100)
	*Entamoeba coli*	-	23 (57.5)	5 (16.66)	-	2 (20)
	*Theileria equi*	1 (5)	25 (62.5)	-	-	4 (40)
	*Trypanosoma evansi*	-	6 (15)	-	-	10 (100)
Arthropoda	*Gasterophilus intestinalis*	18 (90)	40 (100)	30 (100)	19 (95)	10 (100)
	*Gasterophilus nasalis*	-	35 (87.5)	28 (93.33)	10 (50)	7 (70)
	*Boophilus* spp.	-	6 (15)	-	-	6 (60)
	*Hippobosca equina*	-	9 (22.5)	-	-	6 (60)
	*Haematopinus asini*	-	6 (15)	1 (3.33)	-	3 (30)
	*Ctenocephalides felis*	2 (10)	36 (90)	15 (50)	8 (40)	9 (90)
	*Psoroptes equi*	1 (5)	5 (12.5)	-	1 (5)	3 (30)

n=Number of examined donkeys in each governorate

## Discussion

Dealing with helminths, in our study *H. musca*e was recorded in 75% of infected donkeys while in [14-16] recorded 55-90% of donkeys. This indicates that the distribution of this parasite among equines all over the world is quite serious, also indicates the wide distribution of the intermediate host (*Musca domestica*) in Egypt.

In the present study, *D. megastoma* was reported 8.33% while in the other studies performed in different areas of the world, *D. megastoma* was reported in 0.69-47% of donkeys [14,15], other studies did not record *D. megastoma* in any groups of family Equidae [17].

The prevalence of *P. equorum* was 25%, which is less than the results recorded by Shrikhanda *et al*. [17] 29.26 % and 43% recorded by Ayele *et al*. [18]. This may be due to different grazing areas around family Equidae and the lack of awareness about the health of animals in these areas, while the current prevalence of *P. equorum* (25%) was higher than the previous record of 17.3% by Fikru *et al*. [19].

The prevalence of *Strongylus* spp. disagreed with the result of 99.5% [17], 100% by Alemayehu [20], 96.77% by Sinasi [21], and 92% by Ayele and Dinka [22]. The different findings might be due to the differences in the climate, agro-ecological conditions, variation in sample size, and sampling method differences [23]. In addition, this might be associated with donkeys which could be neglected in these areas, kept under poor management conditions, and receiving less attention from owners [24].

Due to the difficulty in the identification and complex taxonomy of cyathostomins, few workers have identified these parasites to the species level in donkeys [25-28]. In the present study, *Cyathostomum* spp. was recorded with the prevalence rate of 91.66%, this is very high and agreed with the work of Getachew *et al*. [29], who found 17 species of cyathostomins in Ethiopian donkeys. This similarity could be regarded to near similarity of agro-ecological conditions in both countries.

The prevalence of *O. equi* was 50%, and this is higher than 8.53% recorded by Shrikhanda *et al*. [17] and 6.4% recorded by Sinasi [21]. This may be due to the differences between the management systems and climatic conditions between the study areas [23].

The low prevalence of hydatid cyst (8.33%) found in this study is agreed with the findings in donkeys at Donkey Sanctuary, UK [30]. This result might be attributed to the sporadic discharge of gravid segments of *E. granulosus* adult in the feces of dogs and donkeys acts as intermediate host of this helminth [29].

The present study indicated that among different types of helminth parasites, *H. muscae*, *Cyathostomum* spp., and *Strongylus* spp. were found to be dominant in the study. With regard to sex, generally, the helminths parasites were found in females more than males. This might be due to the fact that males are less exposed to infection because they tend to be more solitary [31]. In addition, the female donkeys have a higher infestation because they have lower immunity due to gestation, lactation, and stress occurring during this period [32].

It is assumed that sex is a determinant factor influencing the prevalence of parasitism [33]. With regard to age, the highest prevalence of helminths were seen in old age, and this may be due to loss of body conditions and decrease of immunity, whereas the age of the animal increases, the immunity decreases [32], except in harboring by *P. equorum* where this parasite was found in young equines more than older ones. This might have been because the donkeys were too old to harbor this parasite [25]. In addition, *P. equorum* is a problem of young equines as the animals have not yet developed immunity [32].

The prevalence of *Cryptosporidium* spp. in donkeys was 6.6% (8 out of 90). The low prevalence of *Cryptosporidium* spp. agrees with Souza *et al*. [34], Laatamna *et al*. [35], Majewska *et al*. [36], and Sturdee *et al*. [37]. In contrast, high prevalence rate reported between 10 and 31% by Caffara *et al*. [38], Grinberg *et al*. [39], and Wannas *et al*. [40]. In the present study, *Cryptosporidium* spp. was found mainly in young age between 1 and 2 years and became lower at the older age (3-5 years). This finding disagrees with Laatamna *et al*. [35] who found it in age > 3years, and not in young one. The *Cryptosporidium* spp. was found in males and females, but Laatamna *et al*. [35] found only in males.

Our results indicated that the prevalence of *E. leuckarti* in donkeys was 8.33%, which is nearly similar to Studzinska *et al*. [41] (7%) and Ghahfarrokhi *et al*. [42] (7.68%). In our investigated data that disagreed with Wannas *et al*. [40] and Atawalna *et al*. [43] recorded high prevalence of 10.71% and 10.3%, respectively, but Nakayima *et al*. [44] found that infection rate was 3.58% and Souza *et al*. [34] found the prevalence of *E. leuckarti* lower than 1%. In our investigations, six donkeys, from 3 to 5 years old, were infected, and four animals were 6-8 years old. This result disagreed with the presence of *E. leuckarti* more in young one as Souza *et al*. [34], but Ghahfarrokhi *et al*. [42] found 2 (7.6%), one 2 years old and another 10 years old. Moreover, in our study, infection by *E. leuckarti* was found in 8 males and 2 females. This agreed with Ghahfarrokhi *et al*. [42] who found in male and female, but Souza *et al*. [34] found it in females only. These differences may be due to geographical variations and various ages and coproscopy methods used [41].

*Sarcocystis* spp. was isolated from 66.66% (80/120) of the donkeys. This result disagreed with Fukuyo *et al*. [45] (93.0% in horses). The rates of detection in the esophagus, tongue, diaphragm, and heart were 80%, 97%, 40.0%, and 14%, respectively. The rates of detection of infection in relation to age are as follows: 1-2years old 17%, 3-5years old 45%, and 6-8years old 50%. The distribution of the *Sarcocystis* spp. in esophagus, tongue, diaphragm, and heart muscle was positively correlated with horse age. The infection with *Sarcocystis* spp. was increased with increasing of age [45].

In our results, *B. coli* infection in donkeys was 91.66%. These results disagree with Wannas *et al*. [40] and Khan *et al*. [46] who found infection 17.85% and 18.3% in donkeys, respectively. While *E. coli* infection in our results was 25%, which is nearly similar to Dissanayake *et al*. [47] in horses (28.8%). This result disagreed with Wannas *et al*. [40], who recorded 3.57% in donkeys.

The hemoparasites seen on microscopy were *T. equi* and *T. evansi* at low parasitemia in 16.66% and 13.33%, respectively. These findings disagreed with Mushi *et al*. [48], who had seen the only hemoparasite to be *T. equi* in 26.8% of the donkeys. However, Atawalna *et al*. [43], observed the only blood parasite was *Trypanosoma* spp. (3.33%). While Gizachew *et al*. [49], revealed that 54 donkeys (13.7%) were positive for piroplasmid merozoites. *T. equi* and *B. caballi* were detected in 48 (12.2%) and 7 (1.8%) samples. Mekibib *et al*. [50] found only 1.3% and 0.5% of donkeys to be infected with *T. equi* and *B. caballi*, respectively, whereas Tefera *et al*. [51] found 2.1% and 1.0% of infected donkeys. Low prevalence data can be caused by false-negative results that may occur due to low parasitemia, especially in the late phase of infection. In this case, polymerase chain reaction carried out with ethylene diamine tetraacetate blood would give a higher prevalence. Another possibility is the *in vitro* cultivation of piroplasms in suspicious blood samples [52].

The different findings between our studies and other works might be due to the differences in the climate, agro-ecological conditions, variation in sample size, and sampling method differences. In addition, this might be associated with donkeys which could be neglected in these areas, kept under poor management conditions, and receiving less attention from owners. In our study revealed most donkeys harbor *G. intestinalis* and *G. nasalis* with high infestation with *Ctenocephalides felis*. The predominant consequence of *G. intestinalis* almost as the prevalence recorded by Hilali *et al*. [53] who recorded 98.3%, this might be because of progress in climatic condition from 1987 to 2017. Our conclusion on *Gasterophilus intestinalis* infestation in Egypt is similar as Otranto *et al*. [54] who recorded two bimodal of life cycle during the year; in April and August, so the grown-up fly recorded in this time. A low predominance rate of 9.9% was recorded by Hoglund *et al*. [55], 43% in Ireland [56], 53% in England [57]. While 95.2% [54] and 94% [58] were recorded in Italy; low prevalence recorded as 2.25% in Germany [59], 0.72% in Ethiopia [60]. While 100% [29] and 28.57% [61] infestation with *G. intestinali*s and *G. nasali*s were recorded in Turkey So; Gasterophilosis was predominant around the Mediterranean area and all over the world Along these lines, *G. intestinalis* is the dominating bot fly in donkeys in Egypt. Other recent similar works in Egypt in specific bot fly in donkeys (*Rhinoestrus* spp.) were carried out by Hilali *et al*. [62], who recorded 100% prevalence in Egypt all over the year.The prevalence rate was 86.6% in 39 inspected horses [63]. It was nearly similar to our study. Ticks in our study (10%) transmit the theileriasis. Tick carries infections worldwide with significance diseases, influencing people, and animals [64].

In this way, in Egypt, this information has updated result of Hilali *et al*. [53]. Thus, this information is important in the therapeutic care of donkeys and also equines. The widespread of *G. nasalis* in the present examination was like the most records of investigations [65,66].

## Conclusion

The present study revealed that donkeys harbor different helminths, protozoal, and arthropods species with prevalence (100%) with single or mixed infection. A detailed study of pathogenicity, treatment, and control strategies of each parasitic species is recommended with periodical treatment of such diseased animals.

## Authors’ Contributions

MMA, MMK, and MTA: Conception, design, and collection of the study. MMA, MMK, and MTA: Analysis and interpretation of the data. MMA, MMK, and MTA: Drafting and revising the manuscript critically for important intellectual content. All authors have read and approved the final manuscript.
